# 3D Printing of Paracetamol Suppositories: An Automated Manufacturing Technique for Individualized Therapy

**DOI:** 10.3390/pharmaceutics14122676

**Published:** 2022-12-01

**Authors:** Vanessa Domsta, Julius Krause, Werner Weitschies, Anne Seidlitz

**Affiliations:** 1 Institute of Pharmacy, Biopharmaceutics and Pharmaceutical Technology, University of Greifswald, Felix-Hausdorff-Str. 3, 17489 Greifswald, Germany; 2 Institute of Pharmaceutics and Biopharmaceutics, Heinrich-Heine-University Düsseldorf, Universitätsstr. 1, 40225 Düsseldorf, Germany

**Keywords:** 3D printing, additive manufacturing, suppositories, pharmaceutical compounding, individualization

## Abstract

Pharmaceutical compounding using the molding technique is the currently applied method for the on-demand manufacturing of suppositories and pessaries. Potential errors of this method are difficult to detect, and the possibilities of individualization of size and shape of the suppositories are limited. In this study, a syringe-based semi-solid 3D printing technique was developed for the manufacturing of suppositories in three different printing designs with the suppository bases polyethylene glycol (PEG) and hard fat (HF). The 3D printed suppositories were analyzed for their visual appearance, uniformity of mass and content, diametrical dimension, breaking force and release behavior and compared to suppositories of the same composition prepared by a commonly used molding technique. The results showed no adverse properties for the 3D printed suppositories compared to the molded ones. Moreover, the easy adaptation of shape using the 3D printing technique was demonstrated by the printing of different sizes and infill structures. Thus, 3D printing has great potential to complement the available manufacturing methods for compounded suppositories, as it represents an automated system for the individualized manufacturing of suppositories that meet patients’ needs.

## 1. Introduction

Suppositories and pessaries are commonly used dosage forms that are applied rectally or vaginally, respectively. Their application is intended for the local or systemic treatment of several diseases, for example, as a laxative, hemorrhoids therapeutic, antimycotic, antiemetic, analgesic, or antipyretic. This route of administration is an advantageous alternative to peroral applications, when those are not suitable due to the absence of ability or willingness to swallow medications, which is often the case with pediatric or geriatric patients or in patients with certain conditions such as unconsciousness or nausea. Further convincing aspects of suppository applications include the relatively constant environmental conditions of the rectum, the improved drug stability due to the absence of gastric enzymes, the prevention of irritation of the gastric mucosa by irritant drugs, and the partial avoidance of hepatic first-pass effect [1,2].

However, suppository therapy tailored to patients’ individual needs has not been widely addressed so far. We recognized a need for personalization in the field of suppositories, for example, in drug dose or shape adjustments. This is particularly relevant in pediatric therapies, but the needs and preferences of each individual patient should also be considered. At the moment, most suppositories weigh 2 g for adults and 1 g for children, and doses for children are adjusted from adult doses considering their body weight [3]. In consideration of the highly differing rectal dimensions and surface areas of children of different ages [3], a higher degree of individualization would be preferable. There are rather uncommon forms of suppositories on the market that enable higher dose flexibility by a dividable scored stick-shape [3]. A more common practice of parents and physicians is the manipulation of suppositories by dividing to achieve appropriate doses for children. Different studies demonstrated a relatively uniform drug distribution in most of the tested commercial suppositories, but the applied method for dividing, for example, in the horizontal, longitudinal, or diagonal direction is not consistent, and only poor accuracy regarding the targeted dose was achievable [4,5,6,7]. Therefore, this practice should only be performed with care, and alternative approaches to individualize doses should be developed.

Moreover, studies on the willingness to administer vaginal suppositories (pessaries) of different shapes, sizes, colors, and firmnesses identified women’s preferences in these parameters, which are associated with the expected quality of handling, comfort in the body, and effectiveness [8,9,10,11]. This highlights the importance of the appearance of these dosage forms for patient adherence and consequently for therapeutic success.

Nowadays, the compounding of customized medicines in pharmacies or hospitals provides a possibility to comply with individual patients’ needs. Compounding in general is applied for reasons of unavailability of a commercial product regarding the dose, drug combination, or an appropriate route of administration, as well as in cases where certain excipients cannot be used for the individual patient due to allergies or dietary restrictions. Moreover, lower costs for some products or occurring shortages may be reasons for compounding [12,13]. For the compounding of suppositories, molding can be applied. The molding technique generally involves the following procedures: preparation of the raw materials, mixing the drug with the melted suppository base, pouring the mass into pre-formed blisters or metallic molds, and subsequent solidification of the suppositories [3]. With this method, an adaption of the drug amounts to the required dose is easily feasible, but the size and shape of the suppositories are limited by the available molds. For example, suppositories with masses of less than 1 g cannot be manufactured due to the lack of smaller metallic molds [3].

Furthermore, compounded medicines are regulated differently by government agencies and have not been clinically evaluated for safety and efficacy as is the case for commercially available products. Therefore, and due to the complex manufacturing technique, compounding errors may also appear in extemporaneously prepared suppositories, which might not be detected by simple tests on visual appearance and uniformity of mass. Studies of Mahaguana et al. [14] on the content uniformity of progesterone vaginal suppositories recorded inconsistent results from pharmacy to pharmacy. Some tested suppositories were subpotent, superpotent, or had varying drug levels within the batch. Kalmár et al. [15] demonstrated the importance of strict adherence to the correct manufacturing technique to achieve uniform drug doses in each suppository. They demonstrated that the drug content of suppositories prepared by pharmacies with predefined intentional errors did not conform to the requirements and exhibited substantial deviations or fluctuations from the target drug dose. Several difficulties are conceivable regarding the manufacturing process of the compounded suppositories. In the beginning, special focus should be on the calculation of the needed amount of suppository base and drug by using the correct displacement factor and molding excess [15]. During processing, a suitable stirring rate affects the resulting quality of the suppositories. Non-uniform drug distribution may be caused by ineffective slow stirring, whereas too intensive stirring may result in decreased suppository weights due to air bubbles or sedimentation of the drug facilitated by reduced melt viscosity as a consequence of shear stress [15]. In addition to an inadequate stirring rate, sedimentation of the suspended drug may be caused by a too high temperature during the pouring process [3,15]. Hence, molding of suppositories should always be performed at the lowest possible temperature. Periodical training is preferable to minimize the risk of compounding errors but is challenging for pharmacies with infrequent prescriptions for extemporaneously prepared suppositories. Good reproducibility as well as nondestructive in-process controls assuring the quality of the finished product are desirable for those small-scale preparations and may potentially be achieved when using alternative manufacturing methods.

3D printing, also referred to as additive manufacturing, is a modern manufacturing technique that has gained increasing interest in the field of pharmaceutical and medical applications. Different 3D printing methods are based on diverse techniques, such as extrusion or laser-assisted procedures, to build three-dimensional objects, but all provide an extensive degree of freedom regarding the construction of desirable shape designs. This advantage was already applied to the production of several pharmaceutical dosage forms, for example, simple to complex tablets, capsules, or implants with an easily adjustable shape or drug dose [16,17,18].

Moreover, researchers have already employed the 3D printing technique to address the topic of suppositories by preparing customizable suppository molds [19,20] or self-supported suppositories as a self-emulsifying system containing dissolved drugs [21,22,23]. Additionally, polyvinyl alcohol has been used to print suppository shells, which further enabled the combination of drugs in one suppository or control the drug release behavior by the used shell design, e.g., multi-layered or equipped with openings [24,25,26].

The aim of this study is the usage of 3D printing technology for the development of an automated manufacturing process for customizable suppositories with reproducible quality and simplified handling to meet individual patients’ needs. For this purpose, a semi-solid 3D printing process was established based on the extrusion of the melted suppository masses from a syringe for two standard suppository bases, namely, a water-soluble polyethylene glycol (PEG) base, and a lipophilic hard fat (HF) base. The antipyretic and analgesic drug paracetamol, also named acetaminophen, was exemplarily incorporated in dissolved and suspended manners. The 3D printed suppositories of different sizes and internal structures were prepared and characterized. Additionally, suppositories prepared by the molding technique were similarly characterized for comparative purposes.

## 2. Materials and Methods

### 2.1. Materials

Hard fat (HF, Witepsol^®^ W25, Caesar & Loretz GmbH, Hilden, Germany) and a mixture of polyethylene glycols with an average molecular mass around 400 and 6000 g/mol (PEG 400, Fagron GmbH & Co. KG, Barsbüttel, Germany and PEG 6000, Merck KGaA, Darmstadt, Germany) were used as suppository bases. The antipyretic and analgesic drug paracetamol (Caesar & Loretz GmbH, Hilden, Germany) was chosen as a model drug due to its established application in the treatment of fever and pain as suppositories, especially in pediatrics. Potassium dihydrogen phosphate and sodium hydroxide were purchased from neoFroxx GmbH (Einhausen, Germany) and AppliChem GmbH (Darmstadt, Germany), respectively.

### 2.2. Methods

#### 2.2.1. Manufacturing of Suppositories by Molding Technique

Suppositories of the same composition (Table 1) as used in the 3D printing process were prepared by a trained specialist using a conventionally applied molding technique. In addition, drug-free suppositories were prepared identically as reference material for analytical procedures. Since paracetamol is soluble in PEG, but not in HF, and these suppository bases have different melting ranges, the procedure for the manufacturing had to differ for both compositions.

**Figure 1:**
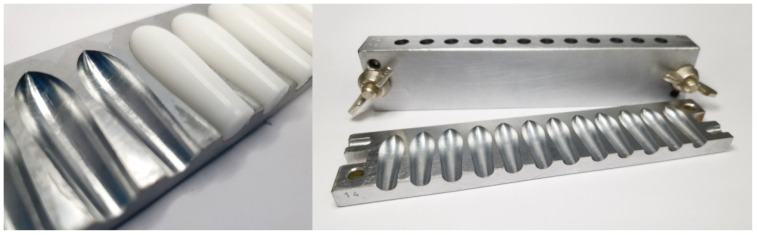
Metallic suppository mold with suppositories of hard fat.

For the manufacturing of PEG-based suppositories, a mixture of PEG 6000 and PEG 400 (75 parts + 25 parts) was melted on the water bath at approximately 70 °C and stirred with 10% (*w*/*w*) paracetamol to a clear melt without any air bubbles. This melt was carefully poured into a metallic mold with torpedo-shaped holes (Figure 1) to avoid air inclusions. After hardening at room temperature, the suppositories were removed from the mold.

In cases of HF formulations, the temperature of the water bath was set to approximately 40 °C. After homogeneous incorporation of 10% (*w*/*w*) paracetamol in the melted HF, the mixture was gently stirred at room temperature until a creamy viscosity was achieved in order to prevent fast sedimentation of the suspended drug particles before pouring the mixture in the metallic mold (Figure 1). Additionally, gently stirring was performed during the pouring procedure after every two filled suppositories.

Drug-free species of PEG and HF were prepared identically without the addition of paracetamol.

#### 2.2.2. Manufacturing of Suppositories by the 3D Printing Technique

The 3D printing technology used is based on the extrusion of softened suppository compositions from a syringe and a cannula heated in the 3D printer into the intended suppository shape by predefined deposition paths. This methodology includes as main steps the preparation of syringes filled with the compositions of the suppository base and drug, the virtual designing of the suppositories, and the actual printing process with the 3D printer using the previously prepared syringes. The individual processes are described in more detail in the following sections.

##### Preparation of the Filled Syringes

Syringes (Mediware Disposable syringes with Luer-Lock, 10 mL, servoprax GmbH, Wesel, Germany; sealed with universal closing Stopper, Combi-Stopper, B. Braun Melsungen AG, Melsungen, Germany) filled with mixtures of the drug and the suppository bases (Table 1) were prepared as feedstock material for the 3D printing process.

For PEG formulations, a mixture of PEG 6000 and PEG 400 (75 parts + 25 parts) was melted on a water bath at 70 °C with 10% (*w*/*w*) paracetamol, in the same way as used for the molding technique. Subsequently, the clear melt was poured into 10 mL syringes via the opening for the temporarily removed piston, and air bubbles were removed before solidification. A second heating step of the syringe was applied if the consistency of the already slightly cooled mixture did not allow the complete removal of all air bubbles. The filled syringes were cooled down at room temperature and were stored until the 3D printing process.

Since the used amounts of paracetamol did not dissolve in the HF melt, the preparation of those syringes required additional effort to guarantee uniform distribution of the drug in the suppository base. For this purpose, HF pastilles and 10% (*w*/*w*) paracetamol were filled directly into 10 mL syringes in a layered manner, with paracetamol located in the center. The sealed syringes were heated to approximately 37 °C in a water bath. The melted mixture was homogenized by intensive shaking. Then, the syringes were gently rotated and air bubbles removed when the viscosity of the melt became a creamy consistency. The final cooling was performed by motorized horizontal rotation of the syringes in contact with cooling accumulators at 10 rpm until complete solidification of the infilled mass. The prepared syringes were also stored at room temperature until the 3D printing process.

##### Design of 3D Printed Suppositories

**Figure 2:**
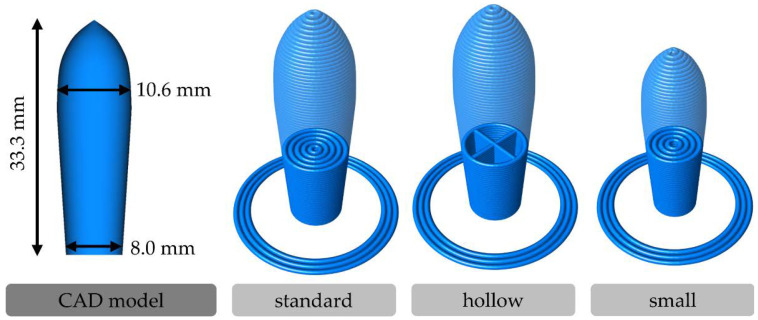
CAD model with dimensions of reconstructed torpedo-shaped suppository and schematic images of the “standard”, “hollow”, and “small” 3D printing designs of the suppositories.

Three different designs (Figure 2) were created with different sizes or internal structures to examine the possibilities of personalization and dose adaption by the application of 3D printing for suppository manufacturing.

The “standard” design is based on the external contour of the torpedo-shaped holes in the metal mold used in the molding technique. A digital reconstruction of this shape was modeled using the computer-aided design (CAD) software FreeCAD 0.18 (https://www.freecad.org, released on 26 October 2019). These “standard” suppositories were printed completely filled with concentric printing lines. The “hollow” design has the same external shape but consists of only one printing line for the contour and single cross-shaped lines as an internal structure. Four air-filled sections are inside these suppositories terminated by two bottom layers. Furthermore, the “standard” design was downsized to 82.7% of the size in each orientation axis to generate a “small” design. The required material amount for these completely filled “small” suppositories should be quite similar to that of the “hollow” suppositories.

##### Semi-Solid 3D Printing Technique

**Figure 3:**
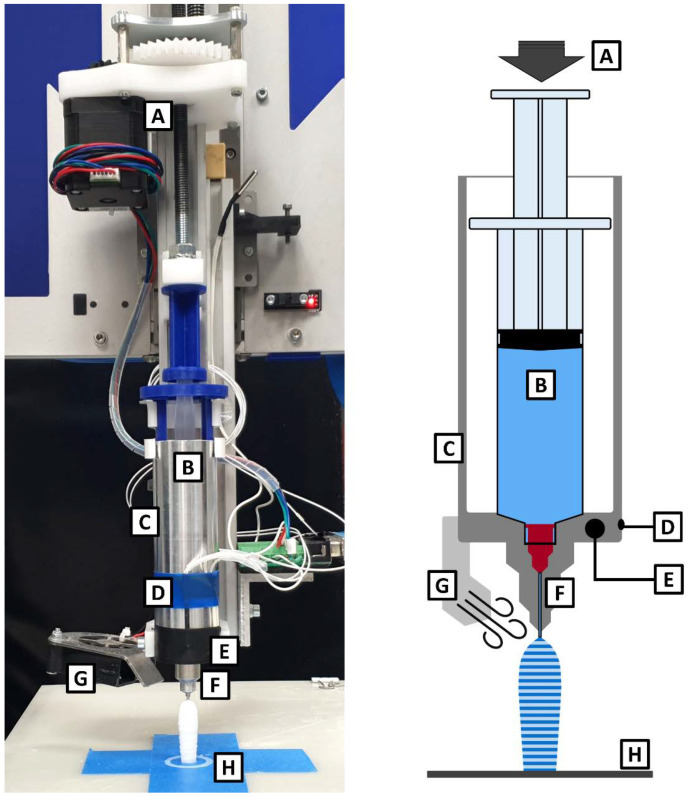
Photographic and schematic image of the 3D printer equipped for semi-solid 3D printing: (**A**) force on syringe piston applied by a motorized threaded rod, (**B**) 10 mL syringe filled with drug-loaded suppository base, (**C**) heatable metal cylinder, (**D**) thermistor, (**E**) heat cartridge, (**F**) heated nozzle, (**G**) cooling fan, (**H**) printing plate equipped with blue tape.

A semi-solid printing technique at moderate temperatures was used for the 3D printing of the different suppository designs. For this purpose, a 3D printer usually used for the filament-based fused deposition modeling printing technique (Multirap M420, Multec GmbH, Illmensee, Germany) was equipped with a heatable metal cylinder (modified Choco 3DRAG, Futura Group srl, Gallarate, Italy) for the insertion of syringes with a volume up to 50 mL (Figure 3).

The previously prepared syringes containing paracetamol in PEG or HF were heated inside the metal cylinder to reach a flowable, but still highly viscous consistency. To achieve these certain consistencies for both compositions, the 3D printer firmware had to be modified to enable the input of decimal values for the target temperatures instead of the integers commonly used. A motorized threaded rod was used to transfer the required force to the syringe piston and extrude the mass through a nozzle with an inner diameter of 0.95 mm (cannula, Sterican^®^ 18 G × 1½″, B. Braun Melsungen AG, Melsungen, Germany; manually shortened to a length of 15 mm). The extruded strands were deposited on a printing plate equipped with blue painter’s tape (3M Corp., Maplewood, MN, USA) and formed the desired shape layer-by-layer by movements of the printing plate (*x*- and *y*-axis) and the syringe print head (*z*-axis). Continuous cooling of the extruded mass by an additionally mounted fan enabled fast solidification.

All required information for the printing process, including, for example, printing routes, temperature, and extrusion amounts, were generated as G-Code by Cura slicing software (version 4.10.0, Ultimaker B.V., Utrecht, The Netherlands). Additional manual changes were performed on the G-Code to specify the start and end procedures as well as nozzle purging behavior before each printing process. The program Repetier-Host (version 2.1.6, Hot-World GmbH & Co. KG, Willich, Germany) was used to control the 3D printer, which enabled the monitoring of temperatures registered by the thermal sensor during the printing process.

During method development, several printing parameters such as printing temperature, speed, or extrusion rate were varied to identify appropriate conditions for each formulation. The final parameters are listed in Table 2.

#### 2.2.3. Characterization of Suppositories

##### Visual Appearance

The quality of each suppository was visually inspected directly after the manufacturing process. Photographic images were exemplarily taken, and microscopic imaging was obtained using a reflected light microscope (Zeiss Stemi 2000-C with Zeiss CL 1500 ECO, AxioCam ICc 1 and AxioVision software v4.8.2, all Carl Zeiss AG, Oberkochen, Germany). Further imaging of the suppository morphology was performed for PEG formulations by scanning electron microscopy (SEM; Phenom FP 3950/00, L.O.T.-Oriel Laser Optik Technologie GmbH und Co. KG, Darmstadt, Germany) with an acceleration voltage of 5 kV. Prior to the SEM examination, the samples were coated with gold–palladium under vacuum using a mini sputter coater (SC7620, Quorum Technologies Ltd., East Sussex, UK).

Furthermore, the printing process for samples of each suppository design was terminated at a height of 15 mm to obtain insights into the internal structure of the suppositories without the necessity to destroy the suppositories, which may be associated with structural changes.

##### Breaking Force

The maximal force that can be applied to the suppositories before breaking was determined on the second day after the manufacturing date using a standard tablet hardness tester (Erweka TBH 225 D, Erweka GmbH, Heusenstamm, Germany) at room temperature. Storage was performed for each suppository individually in a small plastic container at room temperature under the exclusion of light. The suppositories were orientated in a manner such that the force of the jaws acted diametrically on the thickest part of the samples and thus alongside the printing lines, which is apparently the most sensitive area of the printed suppositories.

##### Uniformity of Mass, Diameter, and Drug Content

Twenty samples of each suppository design were weighed (Sartorius analytic A 200 S, Sartorius GmbH, Göttingen, Germany) individually to determine the uniformity of mass by calculating the mean mass and standard deviation (SD). Due to the all-over curved contours of the suppository shape, only the maximal diameter of six samples was measured with a tablet hardness tester (Erweka TBH 225 D, Erweka GmbH, Heusenstamm, Germany) to control the actual dimensions compared to the specifications of the modeled design. The drug content of individual suppositories and the drug distribution within the particular suppository as well as within the syringes were investigated by determining the incorporated amount of drug of six individual suppositories, each cut in half into a tip and tail part with a maximum mass loss of 0.2% due to the division. The division was performed using a scalpel at an approximate height intended for volumetric halving. The exactly calculated height for equal division was previously determined from the CAD model at a height of 16.74 mm for tall and 13.82 mm for small suppositories measured from the bottom touching the build plate. Since the exact division was not feasible, the determined drug content was related to the mass of the tip or tail part, respectively. The tested samples were manufactured from at least three different syringes and from different positions of the printing sequence of each syringe.

For the determination of the paracetamol content, tip or tail samples of PEG formulations were dissolved in approximately 40 mL of demineralized water by agitating at 200 rpm and room temperature in a horizontal shaker (KL-2, Edmund Bühler GmbH, Bodelshausen, Germany). After complete dissolution, the sample solutions were diluted to paracetamol concentrations appropriate to the linear calibration range previously analyzed (1.5–15 mg/mL) and were measured spectrophotometrically (Cary^®^ 50, Agilent Technologies, Inc., Santa Clara, CA, USA; 10 mm quartz cuvette) at a wavelength of 243 nm.

The paracetamol of HF-based samples was extracted for 2 h in 1 L of preheated demineralized water at approximately 60 °C in a horizontal shaker (IKA^®^ KS 3000i control, IKA^®^ Werke & CO. KG, Staufen, Germany) at 50 rpm. This high temperature was selected to ensure fast melting of the HF. Furthermore, an extraction volume of 1 L was chosen, multiple times larger than the suppository volume, to minimize the remaining amounts of paracetamol in the lipid phase of melted HF at the end of the extraction. After resolidification of HF by cooling in a water–ice bath, the solution was filtered through a cellulose filter (Rotilabo^®^ Type 111A, retention range of 12–15 µm, Carl Roth GmbH + Co. KG, Karlsruhe, Germany), diluted, and measured spectrophotometrically in the same manner as PEG samples. Regarding the risk of sedimentation of the suspended paracetamol in HF during the syringe preparation, the drug distribution in four syringes was examined by determining the drug content of the filled mass cut into six slices. The extraction method for HF-based suppositories was additionally performed for six HF samples with an exactly known amount of paracetamol to quantify the extractable drug using this method. For this purpose, HF and 10% (*w*/*w*) paracetamol were weighed into an individual blister cavity, heated, and subsequently shaken until solidification.

To avoid falsified results due to the presence of the suppository bases, PEG and HF, during analytical measurements, molded drug-free suppositories were processed identically, and UV absorbance of the pure bases was determined. These absorbances could be neglected for the final calculations since the measured absorbances for pure suppository bases were less than 0.5% of the lowest absorbance level measured for paracetamol containing suppositories. The absolute drug amounts per suppository were calculated by summing the results of the corresponding parts of the tip and tail.

##### Drug Release Studies

Drug release studies of the paracetamol containing suppositories, manufactured by molding and 3D printing in three different designs, were performed in a paddle apparatus (USP apparatus 2; PT-DT70, Pharma Test Apparatebau AG, Hainburg, Germany) in 900 mL phosphate buffer pH 7.4 (USP) at 37 °C and 50 rpm. A helical sinker of stainless steel was used to prevent the initial floating of the fatty or hollow suppositories. The self-made sinkers were formed of a 0.8 mm thick wire using a 3D printed negative form to achieve repeatable dimensions. The released drug amounts were measured spectrophotometrically every minute using a fiber optic system (wavelength: 243 nm, slit width: 2 mm; Cary 60, Agilent Technologies, Inc., Santa Clara, CA, USA). In addition, measurements were performed identically with drug-free suppositories to ensure that the analytical system was not affected by the PEG and HF suppository base materials.

## 3. Results

Suitable parameters were developed for a reproducible 3D printing process for the manufacturing of paracetamol suppositories based on PEG as well as HF without the need for any additives. The semi-solid 3D printing technique enabled the manufacturing of “standard”, “hollow”, and “small” suppositories with identical printing parameters for each suppository base, performed at set printing temperatures of 45.5 °C for PEG and 29.3 °C for HF. The actual temperatures of the melted mass inside the syringe were expected to be higher since the thermal sensor for temperature control was located on the external surface of the heatable cylinder, and temperatures of approximately 55.6 °C or 34.2 °C were exemplarily measured on central inner surfaces.

### 3.1. Visual Appearance

**Figure 4:**
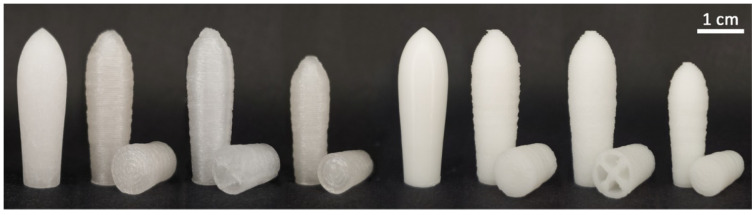
Photographic images of molded and 3D printed suppositories containing 10% (*w*/*w*) paracetamol in the frontal view and internal view of partially printed suppositories. From left to right: PEG mold, PEG “standard”, PEG “hollow”, PEG “small”, HF mold, HF “standard”, HF “hollow”, HF “small”.

Figure 4 presents an overview of the visual appearance of the manufactured paracetamol containing suppositories in total as well as the internal structures of 3D printed suppositories. 

The typical torpedo shape was achieved for suppositories manufactured by molding and 3D printing technique. The suppositories based on PEG had a whitish translucent appearance with a shiny surface, whereas those based on HF were opaque and white-colored. Molded suppositories had a smooth surface except the almost imperceptible seam resulting from the mold halves. In contrast, the surfaces of all 3D printed suppositories were textured by the typical printing layers. Most of these printing lines were deposited homogeneously in parallel lines within the desired external shape.

**Figure 5:**
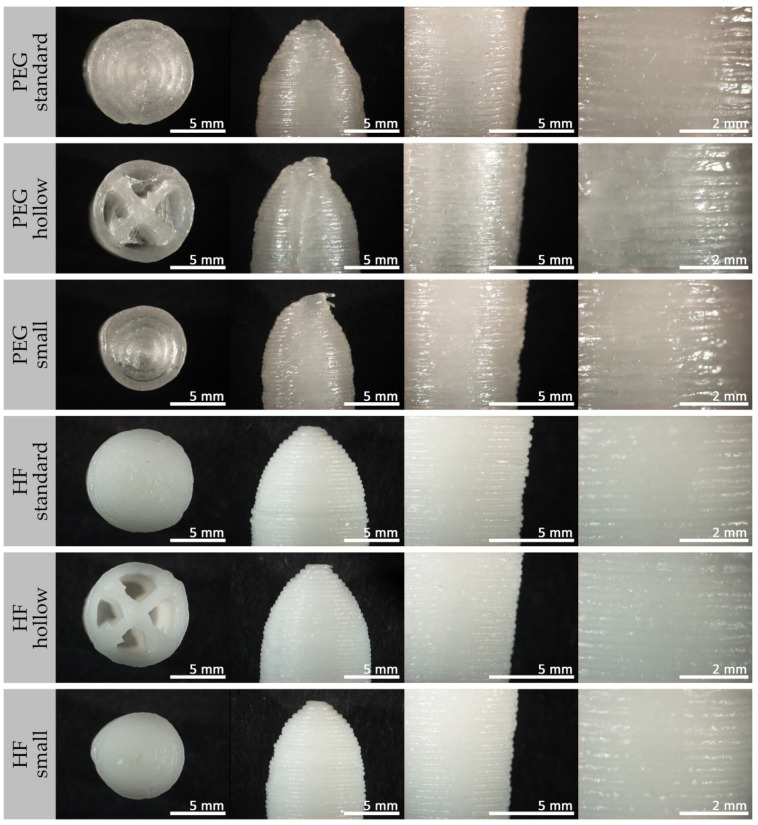
Microscopic images of 3D printed suppositories containing paracetamol based on PEG or HF in different printing designs.

**Figure 6:**
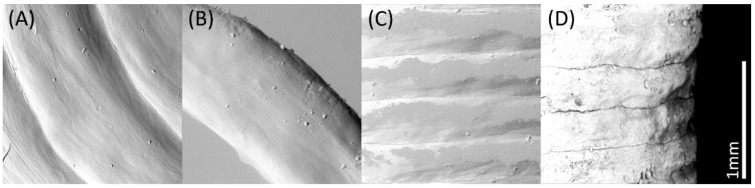
Scanning electron microscopic image of 3D printed suppositories based on PEG containing paracetamol. Top view of printed layers of completely filled printed “standard” suppository (**A**) and single print line of “hollow” suppository (**B**); frontal view of printing lines (**C**) and their contour (**D**).

A detailed view, in particular of the path of the individual printing lines, is very visible in the microscopic and SEM images in Figure 5 and Figure 6.

An interruption of the homogeneous surface of all 3D printed suppositories was visible at the starting point of each new layer, resulting in a vertical seam (compare Figure 5, PEG “hollow”, tip image). In addition, the outer points of the internal lines of the hollow suppositories, each representing a starting and an end point, were slightly thickened, and parts of the material were wiped into threads due to the interrupted extrusion with simultaneous rapid movements of the print head. A shift in the final printing layers at the tip of the suppositories was very recognizable for PEG suppositories but only minimally expressed for HF suppositories.

### 3.2. Breaking Force

**Figure 7:**
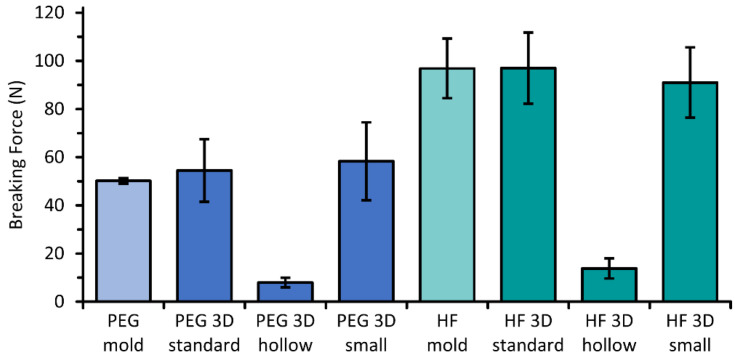
Measured forces that are needed to break molded and 3D printed suppositories containing paracetamol on the base of PEG or HF; mean ± SD, n = 6.

The completely filled suppositories of PEG and HF withstood similar forces before fracturing with values of 50–58 N and 91–97 N on average, respectively, independently of their size or manufacturing technique by molding or 3D printing (Figure 7). Lower forces of 8 ± 2 N or 14 ± 4 N induced the breakage of the “hollow” suppositories. Nevertheless, all suppositories including the “hollow” suppositories were easy to handle, and no care had to be taken during all performed tests.

### 3.3. Uniformity of Mass, Diameter, and Drug Content

The results of the determination of suppository masses and diameters (Table 3) indicated a slightly higher variability of the 3D printed suppositories compared to the molded suppositories, but the standard deviation values were still in a low range for all. The suppositories of the “mold” and “standard” batches as well as of the “hollow” and “small” suppositories reached similar values of mass for each material, as intended in the design. Related results were achieved for the determination of the paracetamol dose in each suppository. PEG suppositories, manufactured by the molding technique, showed the smallest standard deviation of 0.2%, whereas molded HF suppositories showed the highest relative standard deviation of 4.0%, and the deviation of the drug dose of the 3D printed suppositories was in between these values.

**Figure 8:**
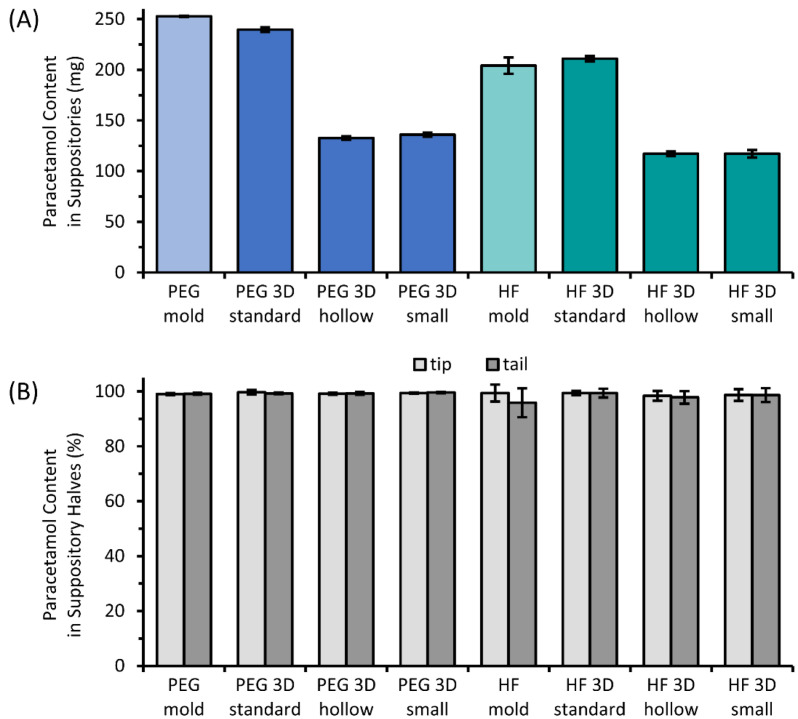
Paracetamol content of the molded and 3D printed suppositories (**A**) based on PEG or HF and the percent drug content in the tip and tail part of those suppositories (**B**); mean ± SD, n = 6.

Figure 8 shows the results of the analysis of drug content in total (Figure 8A) as well as the distribution of paracetamol in the tip and tail of the suppositories illustrated as the percentage of the expected drug amount based on the mass to the respective tip or tail portion (Figure 8B). The drug load of 10% (*w*/*w*) was detected with mean recovery rates in a range of 95.9–99.7% in the tip or tail parts for all batches. Comparable amounts of paracetamol were found in the tip and tail of the tested suppositories. Only a minimal decrease in the mean content was visible for HF suppositories that were manufactured by molding.

The extraction method for HF-based compositions reached a recovery of 98.8 ± 2.8% analyzed for suppositories with exactly known paracetamol content. Similar values of the paracetamol content of 98.8 ± 2.5% were determined for the slices of the prepared syringes with no recognizable trend related to the position in the syringe.

### 3.4. Drug Release Studies

**Figure 9:**
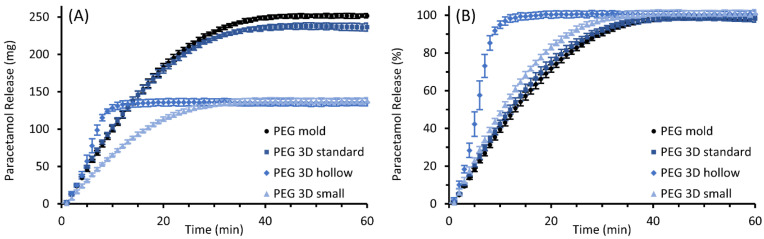
Paracetamol release profiles of molded and 3D printed (“standard”, “hollow”, and “small” design) suppositories based on PEG; mean ± SD, n = 6. Diagram (**A**) shows the cumulative amount of the released drug as mass and diagram (**B**) as the proportion of the expected total amount of 10% (*w*/*w*) paracetamol of the individual suppository mass.

**Figure 10:**
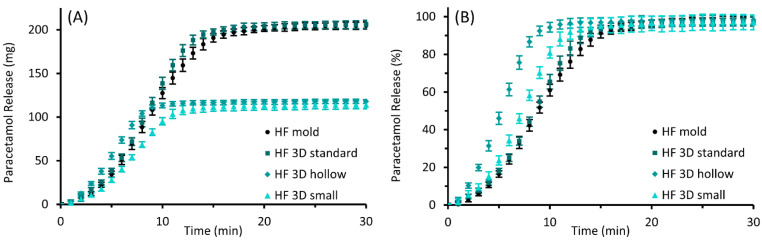
Paracetamol release profiles of molded and 3D printed (“standard”, “hollow”, and “small” design) suppositories based on HF; mean ± SD, n = 6. Diagram (**A**) shows the cumulative amount of the released drug as mass and diagram (**B**) as the proportion of the expected total amount of 10% (*w*/*w*) paracetamol of the individual suppository mass.

The results of the performed in vitro drug release studies are illustrated in Figure 9 for PEG and in Figure 10 for HF suppositories with part A showing the released mass in mg and part B showing the fraction released in % of theoretical drug load.

The paracetamol was released in a relatively constant manner from the completely filled PEG suppositories. The molded and the 3D printed “standard” suppository with the same dimensions showed similar release behavior. The release rates of paracetamol were lower for the 3D printed “small” suppositories, but relative rates were comparable to those of the taller suppositories. These completely filled suppositories released 80% of the drug after 19 to 24 min, whereas suppositories of the “hollow” design released the same proportion faster in only 8 min. Furthermore, a biphasic progression is apparent in the graph of the “hollow” suppositories. The release rate increased after approximately four minutes, while at the same time the inflow of dissolution medium into the hollow structures through the dissolving suppository shell was observed.

The release of paracetamol from the HF suppositories was much faster than from PEG suppositories; 80% of the expected total paracetamol amount was released after 8 to 13 min, with the “hollow” suppositories being the fastest. Furthermore, the profile of released paracetamol masses was quite similar for all suppository designs, and release rates increased after a few minutes.

For drug-free suppositories, only minor background noise signals were detected near the baseline.

## 4. Discussion

This work demonstrates the successful development of a semi-solid 3D printing method for the manufacturing of customizable suppositories as an alternative manufacturing method to the molding processes commonly used in pharmaceutical compounding.

The hydrophilic PEG mixture and the lipophilic HF are common suppository bases, which release their incorporated drugs by dissolving or melting in the body, respectively. Drugs suspended in the suppository base are often beneficial due to their faster drug release from the dosage form but are more challenging during the manufacturing process. This had to be taken into account as well for the development of the 3D printing process. Different conditions were examined with the tested compositions with an exemplary drug load of 10% (*w*/*w*) paracetamol since, the incorporated therapeutic relevant amounts of paracetamol were soluble in PEG but not in HF.

### 4.1. Semi-Solid 3D Printing Process

The term semi-solid 3D printing includes several techniques that build three-dimensional objects by the extrusion of gels or pastes from a syringe through a nozzle. A processable consistency of these semi-solid or semi-molten materials could be achieved by heating at low temperatures or mixing with appropriate solvents, and the extrusion process could be performed by pneumatic systems, electrical impulses, or mechanical systems, including pistons or screws [27].

In this work, the semi-solid 3D printing technique is based on a heatable syringe system combined with a piston, which is also suitable for thermo-sensitive drugs due to its limited working temperatures. The aim was the development of a suitable printing process for two different compositions in three different designs. The process should be automated as much as possible to print suppositories reproducible in different shapes with identical printing parameters, since in-depth process parameter optimization is not possible if on-demand compounding of individualized doses is desired.

During the development of the 3D printing process, the optimal printing temperature was identified as the most important parameter, as only minimal deviations resulted in changes in the quality of printed objects. According to the manufacturer information, HF melts at a temperature of 34 °C, PEG 6000 melts at 57.5 °C, and PEG 400 is fluid at room temperature. Due to these low melting temperatures and the need for a highly viscous melt for printing, the heating temperature had to be set very precisely. Temperatures that are too high increase the risk of drug sedimentation in the syringe in the case of a suspended drug. Additionally, if the material is too liquid, the object might not hold its shape due to the weight of further printing layers or even drip uncontrollably out of the nozzle. Temperatures that are too low may lead to clogging of the nozzle with solidified material. During systematic trial-and-error approaches to find the optimum printing temperature, all these phenomena were observed, as described similarly in the literature [21,28]. The usage of the standard firmware setting with temperature steps of 1 °C did not lead to satisfactory results regarding the melt viscosity, especially for HF suppositories. The sensitive requirements of this small melting range were met by changing the firmware code to decimal temperature inputs, using a cooling fan, and disabling power switching of the heat cartridge between printing runs in the G-Code, which would usually cause higher fluctuations in temperature during the initial printing phase. The melt viscosity might possibly be improved by the addition of gel-forming additives, such as silicium dioxide or HPMC, but this approach was not pursued here to keep the suppository formulation as simple as possible and to exclude further effects on the suppository characteristics [29,30]. External preheating of the syringes in a water bath, as performed in our initial studies on HF printability [31], was no longer necessary due to the exact temperature adjustment. However, the application of preheating slightly below the printing temperature could be beneficial to minimize the time for thermal equilibration of the syringe in the print head and thus optimize printer idle times.

A further important printing parameter was the extrusion rate. The right amount of semi-molten suppository material had to be extruded to achieve constant material strands of the intended thickness. Additionally, the initial pressure of the piston at the start seemed to influence the printing process as well. A nozzle purging procedure was implemented in the beginning of each printing process to ensure the same pressure conditions. A small number of the firstly printed suppositories per syringe needed to be discarded and excluded from further testing because of their higher masses, probably caused by an overextrusion of material due to the manual insertion of the syringe with manual preloading of the piston. Problems like this are easily detectable by performing a simple mass comparison as in process control. Possibly, this issue could be avoided by the use of pneumatic-based systems, as their response time for pressurizing the system is rapid [27]. Alternatively, the nozzle purging could be optimized, or the initial preloading of the piston could be automated to maintain the advantages of the mechanical system, which is simpler, needs no air compressor, and enables fast syringe exchanges [27].

The manufacturing time for a 3D printed suppository is directly dependent on the printing speed. The speed was set relatively low at 5 mm/s due to the sensitive printing process for the used suppository materials. One printing run took approximately 14 min for the “small” or “hollow” and 23 min for the “standard” design, but subsequent cooling was not necessary for further handling, because every layer was already solidified during the printing process. The manufacturing of suppositories by molding is also a comparatively time-consuming process regarding the preparational steps, molding procedure, and the long cooling phase, at least when small batches are produced. The time exposure of both manufacturing processes is not readily comparable, since molding of a higher number of suppositories at once is common, but the 3D printing process works without much personal involvement. Moreover, the effectiveness of the printing process could be increased in the future by the usage of multiple printing heads for the simultaneous printing of a higher number of suppositories than is already applied for printed oral dosage forms [32].

The identical printing parameters for each material enabled the 3D printing of suppositories in three different designs. Especially the “hollow” suppositories represent a challenging printing design due to their single printing lines. Small printing defects cannot be compensated by adjoining supporting lines and may be carried onto further printing layers. The geometrical freedom of 3D printing processes was proven for suppository bases HF and PEG due to the different printable designs. Regarding the enormous flexibility in shape, already shown for 3D printing processes in the pharmaceutical field [16,33], this application shows high potential for the on-demand manufacturing of customized suppositories. Individual adjustments of dose, size, or shape of the suppositories are easily feasible. Even the combination of different drugs or bases in one dosage form, as described in the literature for oral applications [34,35], would be transferrable to the developed process by the addition of printing heads. Meeting the individual patients’ needs by this system could enable better adherence and thus more successful therapies.

### 4.2. Visual Appearance

The visual appearance of the 3D printed suppositories indicates good quality, even for challenging printing sections such as single printing lines, the adherence of the first printing layer on the build plate, and the printing termination in a sharp tip. The resolution of these suppositories is relatively low with a layer width of 0.9 mm and height of 0.4 mm compared to other printing techniques, but not unusual for semi-solid 3D printing [36,37,38]. Since higher resolutions are related to increased processing times, the performed quality seemed to be good for the intended application site, and the time investment is acceptable. However, the pronounced structured surface of the suppositories might irritate patients that are only accustomed to the commonly smooth surface of molded suppositories. Studies on the acceptance of 3D printed oral dosage forms stated the relationship between patients’ preferences and familiar dosage forms [39,40]. Hence, in order to ensure patient acceptance, detailed information needs to be given by the physicians or pharmacists before the first application of the 3D printed suppositories. Nevertheless, the layered surface could provide a beneficial effect on the safety of this dosage form. A loss of uniform drug distribution is risked when suppositories with a low melting temperature are stored in warm environments such as on summer days or inside a car [6]. This implies a high risk of dosing failure by the application of a resolidified and thereafter divided suppository. The resolidification process might not be visible for molded suppositories in foil packaging but is easily recognizable by the patients of 3D printed suppositories.

### 4.3. Breaking Force

The examined values of the breaking force provided information about the hardness of the tested suppositories as an essential aspect if they could withstand acting forces during packaging, transport, and handling. Interestingly, similar forces were needed for rupture of the completely filled suppositories of each composition, including the molded and 3D printed “small” and “standard” suppositories. Thus, the manufacturing process by 3D printing did not seem to negatively affect the mechanical stability of suppositories independent of the adapted size. Due to the internal structure of the “hollow” suppositories, the risk of mechanical damage is higher regarding the lower breaking strengths of those, but handling for testing and imaging was possible without any problems observed. Even though this must be verified in further tests, problems during administration are not to be expected from the results obtained so far.

### 4.4. Uniformity in Mass, Diameter, and Drug Content

The production of the 3D printed suppositories with the intended dimensions of the modeled design was verified by diameter and mass measurements. The measured diameters of the 3D printed suppositories were comparable to the molded ones and comply with the intended dimensions or were only very slightly thinner. Smaller deviations from the CAD dimensions are reasonable by the shrinking of the material during the cooling process, a common phenomenon in 3D printing processes. This shrinking may occur to different extents depending on the printed shape, material, and printing parameters and is especially challenging for thin-walled objects [41,42,43]. A so-called volume contraction is also commonly observed when preparing suppositories via molding. Furthermore, similar masses were achieved for the molded and “standard” as well as for the “hollow” and “small” suppositories, as intended by the design, since two comparable drug doses should be tested in the release studies. Moreover, the masses of the 3D printed objects were well reproducible. The mass deviations were the smallest for the molded suppositories, but values of the 3D printed suppositories were well below the limit of 10% required in the United States Pharmacopoeia (USP) chapter, “Quality Assurance in Pharmaceutical Compounding”. The drug content of 90–100% of the labeled amount defined by the USP monograph for acetaminophen suppositories was met by all tested suppositories under the assumption that the drug content of 10% (*w*/*w*) represents the labeled amount. The safe application, even after dividing the suppositories if this is done with the correct masses of each half, is ensured by the uniform distribution of the drug between all and within each suppository. The obtained uniform drug distribution of 3D printed suppositories is comparable to the results described in the literature for commercial paracetamol suppositories, where no significant difference in the drug content was detected in suppositories of different batches, dosages, and producers divided into two or four parts [5,6]. The tendency of drug sedimentation was minimally observed for molded HF suppositories, which showed slightly higher drug content in the tip than in the tail. The difference is marginal and highlights the achievement of good quality suppositories by molding techniques but also signals the potential risk of sedimentation by inadequate manufacturing. Accordingly, preliminary testing underlined the importance of high-quality syringe preparation with uniform drug distribution. The syringe preparation under non-optimized conditions at too high temperatures and insufficient agitation during cooling resulted in a gradient of drug content inside the syringe and would consequently increase the variability of the individual paracetamol content of the 3D printed suppositories. Since the drug distribution inside the syringes is not visually verifiable, special attention should be paid to the correct preparation and storage conditions. Potentially, prospective syringe preparations could be performed industrially on a larger scale, including a temperature-controlled and trackable system for storage and transport, before being received by the printing operators. Those problematical sedimentation phenomena were negligible for the manufacturing of PEG suppositories due to the fact that the drug dissolved in this suppository base.

Different paracetamol doses per suppository were 3D printed in a range of approximately 113 mg to 240 mg for PEG samples and 117 mg to 211 mg for HF samples by exemplarily adapting the size or inner structures. Doses in between or even in a wider range are certainly obtainable by further sizes, shapes, or inner structures, but they are also limited by their applicability. Adapting the drug load inside the syringe as feedstock material is needed for further enlarging of the printable range or adapting the dose to very high or low ranges, which might be necessary for drugs with other therapeutic concentrations. Homogenously distributed drugs are essential for a successful 3D printing process as well as sufficient stability of the object by the binding properties of the used base material. Semi-solid 3D printing has already been used to process dosage forms with drug loads of only 0.12% as well as extremely high drug loads of 96%, which were obtained in the final state, and accordingly approximately 10% less in the processing state of the printing paste [21,44]. The actual limits have probably to be determined for specific combinations of drugs and excipients, but this technique in general seems to be sufficient for manufacturing a wide range of drug loads.

### 4.5. Drug Release Studies

Most of the suppository release studies described in the literature were performed as quality control methods, and only a few intended to provide predictive information of potential in vivo behavior [45]. A standard dissolution tester can mimic the average temperature and pH value of the target region, but the inclusion of physiologically relevant media volumes, their mixing, viscosities, mechanical stress on dosage forms, surface areas, and transport mechanisms is difficult. The presented study focused on the comparison of 3D printed and molded suppositories. Accordingly, a predictable dissolution method was not intended. Since multiple factors such as the characteristics of test medium, drug substance, and suppository base affect the drug release from suppositories, there is no test method suitable for all formulations [46,47]. Hori et al. demonstrated different results of release behavior by testing the same lipophilic acetaminophen suppositories using basket, paddle, dialysis, and flow-through cell methods at different agitation speeds [48]. In our preliminary experiments performed with the basket method, we observed similarly incomplete drug release profiles with high standard deviations for HF suppositories. Finally, reproducible results were achieved for HF and PEG suppositories by the use of a paddle apparatus in combination with a sinker.

The release mechanism of suspended paracetamol in HF suppositories can be characterized by three main steps after melting of the suppository base at body temperature. Firstly, the drug particles sediment within the suppository base to the interface between lipid and aqueous phases, followed by wetting of the particles by the aqueous phase and finally the dissolution of the drug into the aqueous phase [49]. Regarding these mechanisms, the similar release rates of all HF suppositories can be explained by similar amounts of HF melting per unit time, forming a similar volume-to-interface ratio on the surface of the dissolution medium. The melting process was observed to be completed faster for “small” and even more for “hollow” suppositories, possibly due to their smaller total mass or the thin structure. Consequently, the drug release is completed earlier, and relative release rates seem to be faster than for the taller suppositories. Considering the relatively small temporal differences in the release behavior, the different suppository designs must not be expected to distinctively affect the therapeutic effect in application.

In contrast, the paracetamol incorporated in PEG suppositories was released mainly by dissolution and erosion of the matrix and was released at a lower rate than from HF suppositories. Similar relative release rates were obtained for all completely filled suppositories, explainable by similar volume/surface ratios of the suppositories. However, smaller absolute paracetamol amounts were released per time in the case of “small” suppositories due to their smaller absolute surfaces. The “hollow” suppositories released the drug completely differently than the “small” suppositories despite their same masses. Interestingly, the release rates were similar to those of the “standard” and molded suppositories in the first section of the release profile. During this phase, the outer shell of the suppositories dissolved, which was identical for all three designs. In the following phase, the dissolution medium could flow into the perforated shell of the “hollow” suppositories. This strongly increased the contact area between the suppository and dissolution medium, accompanied by a sudden increase in paracetamol release rates compared to the completely filled suppositories. Similar effects of infill properties on the drug release behavior were already reported for 3D printed oral dosage forms [50,51,52]. Thus, suppository manufacturing by 3D printing enables a variation of release characteristics in addition to the customizable dose adjustment for PEG suppositories. However, it remains to be investigated whether this effect also occurs in vivo, where the amount of freely flowing fluid and the viscosity of the fluid must be expected to greatly differ from the conditions in the dissolution study.

Furthermore, a rupture of the fragile structure of the “hollow” suppositories in vivo is conceivable due to the contractual pressures of the body. The increased surface area of the spread suppository mass after rupture might still have beneficial effects on the drug absorption.

In conclusion, no substantial differences in release behavior were identified between molded and 3D printed suppositories of the same dimensions.

### 4.6. Future Prospects

The concept idea of 3D printed suppositories for the future envisions 3D printers in many pharmacies and hospitals. Syringe cartridges should be industrially manufactured and purchasable with different drugs and suppository bases. Ideally, profiles with optimal printing parameters are accompanied by each syringe, possibly as an integrated chip that is readable by the 3D printer. After simply inserting the syringe into the 3D printer without requiring contact with potentially harmful drugs, the operator can select the suppository shape and drug dosage in software that calculates the required suppository mass and printing paths. Admittedly, technical improvements as well as control mechanisms, for example, implementation of a scale directly in or underneath the build plate or camera monitoring assisted by artificial intelligence, are still needed before this concept can guarantee a simple and safe individualized manufacturing method.

## 5. Conclusions

An automated 3D printing process was successfully developed for the reproducible manufacturing of paracetamol suppositories in three different designs using HF and PEG as suppository bases. The tested properties of the 3D printed suppositories were comparable to molded suppositories. To guarantee the compliance of patients, they need to be well informed about this new dosage form. However, 3D printing can provide a simple and safe alternative manufacturing technique for small-scale manufacturing of suppositories in the future. Moreover, the geometrical freedom of this technology enables the on-demand manufacturing of suppositories that are customizable with respect to drug dose and shape to meet patients’ individual needs.

## Figures and Tables

**Table 1:** Overview of the composition and preparation techniques of the suppository batches manufactured by the molding technique or the 3D printing process (PEG: polyethylene glycol, HF: hard fat).

Name (Design)	Manufacturing Technique	Composition
PEG mold	Molding	PEG 6000 (75 parts) PEG 400 (25 parts) +Paracetamol (10%)
PEG 3D standard	3D printing	
PEG 3D hollow	3D printing	
PEG 3D small	3D printing	
HF mold	Molding	Hard fat +Paracetamol (10%)
HF 3D standard	3D printing	
HF 3D hollow	3D printing	
HF 3D small	3D printing	

**Table 2:** Developed parameters of the 3D printing process.

Printing Parameter	PEG Suppositories	HF Suppositories
Nozzle Inner Diameter	0.95 mm	0.95 mm
Line Width	0.9 mm	0.9 mm
Layer Height	0.4 mm	0.4 mm
Printing Temperature	45.5 °C	29.3 °C
Build Plate Temperature	not heated	not heated
Printing Speed	5 mm/s	5 mm/s
Travel Speed	100 m/s	100 mm/s
Fan Cooling	100%	100%
Build Plate Adhesion	skirt	skirt
Build Plate Material	blue tape	blue tape

**Table 3:** Results of the determination of suppository masses, diameters, and drug doses (paracetamol).

Name	Suppository Mass Mean ± SD, n = 20 (g)	Maximum Diameter Mean ± SD, n = 6 (mm)	Drug Dose Mean ± SD, n = 6 (mg)
PEG mold	2.549 ± 0.010	10.65 ± 0.04	252.7 ± 0.5
PEG 3D standard	2.407 ± 0.035	10.49 ± 0.23	239.6 ± 2.2
PEG 3D hollow	1.344 ± 0.024	10.15 ± 0.09	132.7 ± 1.6
PEG 3D small	1.368 ± 0.018	8.55 ± 0.21	136.0 ± 1.9
HF mold	2.102 ± 0.007	10.57 ± 0.03	204.1 ± 8.1
HF 3D standard	2.122 ± 0.022	10.64 ± 0.16	210.9 ± 2.6
HF 3D hollow	1.192 ± 0.017	10.34 ± 0.10	117.1 ± 2.2
HF 3D small	1.179 ± 0.018	8.65 ± 0.12	117.1 ± 3.8

## Data Availability

The data presented in this study are available upon request from the corresponding author.
